# Geographic remoteness‐based differences in in‐hospital mortality among people admitted to NSW public hospitals with heart failure, 2002–21: a retrospective observational cohort study

**DOI:** 10.5694/mja2.52635

**Published:** 2025-04-20

**Authors:** Imants Rubenis, Gregory Harvey, Karice Hyun, Vincent Chow, Leonard Kritharides, Andrew P Sindone, David B Brieger, Austin CC Ng

**Affiliations:** ^1^ Concord Repatriation General Hospital Sydney NSW; ^2^ The University of Sydney Sydney NSW; ^3^ ANZAC Research Institute Sydney NSW

**Keywords:** Heart failure, Cardiomyopathies, Mortality, Rural health services, Ventricular dysfunction

## Abstract

**Objective:**

To examine associations between remoteness of region of residence and in‐hospital mortality for people admitted to hospital with heart failure in New South Wales during 2002–21.

**Study design:**

Retrospective observational cohort study; analysis of New South Wales Admitted Patient Data Collection data.

**Setting, participants:**

Adult (16 years or older) NSW residents admitted with heart failure to NSW public hospitals, 1 January 2002 – 30 September 2021. Only first admissions with heart failure during the study period were included.

**Main outcome measures:**

In‐hospital mortality, by remoteness of residence (Australian Statistical Geography Standard), adjusted for age (with respect to median), sex, socio‐economic status (Index of Relative Socioeconomic Advantage and Disadvantage [IRSAD], with respect to median), other diagnoses, hospital length of stay, and calendar year of admission (by 4‐year group).

**Results:**

We included 154 853 admissions with heart failure; 99 687 people lived in metropolitan areas (64.4%), 41 953 in inner regional areas (27.1%), and 13 213 in outer regional/remote/very remote areas (8.5%). The median age at admission was 80.3 years (interquartile range [IQR], 71.2–86.8 years), and 78 591 patients were men (50.8%). The median IRSAD score was highest for people from metropolitan areas (metropolitan: 1000; IQR, 940–1064; inner regional: 934; IQR, 924–981; outer regional/remote/very remote areas: 930; IQR, 905–936). During 2002–21, 9621 people (6.2%) died in hospital; the proportion was 8.0% in 2002, 4.9% in 2021. In‐hospital all‐cause mortality was lower during 2018–21 than during 2002–2005 (adjusted odds ratio [aOR], 0.52; 95% confidence interval [CI], 0.49–0.56); the decline was similar for all three remoteness categories. Compared with people from metropolitan areas, the odds of in‐hospital death during 2002–21 were higher for people from inner regional (aOR, 1.12; 95% CI, 1.07–1.17) or outer regional/remote/very remote areas (aOR, 1.35; 95% CI, 1.25–1.45).

**Conclusion:**

In‐hospital mortality during heart failure admissions to public hospitals declined across NSW during 2002–21. However, it was higher among people living in regional and remote areas than for people from metropolitan areas. The reasons for the difference in in‐hospital mortality should be investigated.

**The known**: Each year, more than 180 000 people are admitted to Australian hospitals with heart failure. It is not known whether treatment advances over the past two decades have improved in‐hospital survival for people living outside major cities.

**The new**: During 2002–21, in‐hospital survival for people admitted with heart failure to New South Wales public hospitals improved, but the likelihood of dying in hospital was 12% higher for people from inner regional areas than for people from major cities, and 35% higher for people from outer regional or remote NSW.

**The implications**: Determining the reasons for these differences would enable targeted programs for improving outcomes for people in regional and remote Australia with heart failure.

Cardiovascular disease is the leading cause of death in Australia.^1^ Heart failure, second only to coronary artery disease as a cardiovascular cause of illness and death, is the main reason for 180 000 hospital admissions in Australia each year.^1^ The rates of hospitalisation with and death from heart failure increase with geographic remoteness,^1^, ^2^ but the reasons for the differences, and changes over time, have not been investigated in detail.

Almost one‐third of people in Australia do not live in metropolitan areas,^3^ but only 2.4% of National Health and Medical Research Council funding is for research that specifically benefits people living in regional or remote Australia.^4^ Further, people living in regional and remote areas do not have adequate access to specialist cardiology care, which is very important for both inpatient and outpatient follow‐up care. Treatment of cardiovascular disease guided by a cardiologist is associated with lower risk of death from heart failure.^5^, ^6^ People in regional Australia have less access to specialist heart failure care and are more likely to have general practitioners as their primary heart failure physicians than people in metropolitan areas,^7^, ^8^, ^9^ and their clinical outcomes are poorer.^10^, ^11^, ^12^ The management of heart failure has markedly improved over the past two decades, and updates are regularly published by Australian^13^ and overseas cardiology societies^14^, ^15^ to guide physicians managing patients with heart failure.

Whether the geographic differences in outcomes for people with heart failure in Australia have changed with these improvements is unclear. We therefore examined associations between remoteness of region of residence and in‐hospital mortality for people admitted to hospital with heart failure in New South Wales during 2002–21.

## Methods

Established in 2006, the Centre for Health Record Linkage (CheReL; https://www.cherel.org.au) maintains a large data linkage system for health‐related data for New South Wales residents; it includes hospitals administrative data since 31 July 2001. It includes the Admitted Patient Data Collection (APDC), which includes data for more than 97% of hospital admissions in NSW.^16^ For our retrospective observational cohort study, we identified all admissions with primary diagnoses of heart failure during 1 January 2002 – 30 September 2021, as described previously.^17^, ^18^, ^19^, ^20^ De‐identified data for cases that met the inclusion criteria were provided to the research team by CheReL. We analysed data for NSW residents aged 16 years or older at the time of admission with heart failure to public hospitals. We excluded patients without documented area of residence; for people admitted more than once with heart failure during the study period, we included only the first admission. We excluded private hospital admissions to reduce geographic bias, as most private hospitals are in metropolitan areas. We report our study in concordance with the STROBE guidelines for reporting observational studies.^21^

### Data sources and study outcome

For each heart failure admission, we extracted the admission date, region of residence (Statistical Area Level 2 [SA2] code), age, sex (male, female), hospital length of stay, and whether the person died in hospital (the primary outcome). The primary diagnosis and all background diagnoses were coded in the APDC according to the International Statistical Classification of Diseases, tenth revision, Australian modification (ICD‐10‐AM) (Supporting Information, table 1). The number of medical conditions other than heart failure was quantified using the Charlson comorbidity index (CCI). Socio‐economic status (Index of Relative Socioeconomic Advantage and Disadvantage, IRSAD^22^) was based on SA2 region of residence;^23^ a higher IRSAD score indicates lower disadvantage and greater advantage.

### Statistical analysis

We stratified patients by region of residence (SA2) at the time of heart failure admission. We classified remoteness according to the Australian Statistical Geography Standard, a measure of relative access to services, with five categories;^23^ for this study, we defined three categories: metropolitan (= major cities; the reference group), inner regional, and outer regional/remote/very remote.

We summarise continuous variables as means with standard deviations (SDs) or medians with interquartile ranges (IQRs); the statistical significance of between‐group differences was assessed using one‐way analysis of variance (ANOVA; parametric variables) or Kruskal–Wallis tests (non‐parametric variables). We summarise categorical variables as numbers and proportions; the statistical significance of between‐group differences was assessed in χ^2^ tests. We examined associations of variables with in‐hospital mortality using binary logistic regression; the variables assessed were remoteness category, age (with respect to median), sex, IRSAD score (with respect to median), other diagnoses, hospital length of stay, and calendar year of admission (by 4‐year group, to reduce variability by year); we report odds ratios (ORs) with 95% confidence intervals (CIs). We performed a separate multivariable model that incorporated CCI to assess the impact of morbidity burden on in‐hospital mortality and to assess the robustness of our results. To reduce potential multicollinearity, tolerance was set at greater than 0.4 (ie, variance inflation factor of 2.5). Variables for which *P* < 0.05 were included in the multivariable analysis; we report adjusted ORs (aORs) with 95% CIs. All analyses were performed in SPSS 23 (IBM) and Prism 8 (GraphPad). *P* < 0.05 (two‐tailed) was deemed statistically significant.

### Ethics approval

The NSW Population and Health Services Research Ethics Committee approved the study and waived the requirement for individual consent to use health information (2013/09/479). All patient‐related data were de‐identified and analysed anonymously. The study protocol conformed with the 2013 Declaration of Helsinki.

## Results

Our analysis included 154 853 people admitted to hospital with heart failure during 1 January 2002 – 30 September 2021 (Supporting Information, figure 1); 99 687 lived in metropolitan areas (64.4%), 41 953 in inner regional areas (27.1%), and 13 213 in outer regional/remote/very remote areas (8.5%). The median age at admission was 80.3 years (IQR, 71.2–86.8 years), and 78 591 were men (50.8%); the proportion of men was larger for outer regional/remote/very remote areas (52.2%) than metropolitan areas (50.2%). The most frequent other cardiac conditions were atrial fibrillation or flutter (39 763 people, 25.7%) and ischaemic heart disease (31 949, 20.6%); the most frequent cardiovascular risk factors were diabetes (42 435 people, 27.4%) and hypertension (41 146, 26.6%). The median CCI score for people living in metropolitan areas was 1 (IQR, 0–2), for those in inner regional it was 1 (IQR, 0–2), and for those in outer regional/remote/very remote areas it was 0 (IQR, 0–1). The median IRSAD score was higher for people from metropolitan (1000; IQR, 940–1064) than those from inner regional (934; IQR, 924–981 and outer regional/remote/very remote areas (930; IQR, 905–936) (Box 1).

Box 1 Characteristics of people aged 16 years or older admitted with heart failure to New South Wales public hospitals, 1 January 2002 – 30 September 2021, by remoteness of residence*

**Table:** 

Parameters	All patients	Metropolitan	Inner regional	Outer regional/remote/very remote
Number of patients	154 853	99 687 (64.4%)	41 953 (27.1%)	13 213 (8.5%)
Age (years), median (IQR)	80.3 (71.2–86.8)	80.6 (71.4–87.0)	80.0 (71.2–86.5)	79.0 (70.0–86.1)
Sex (men)	78 591 (50.8%)	50 034 (50.2%)	21 664 (51.6%)	6893 (52.2%)
IRSAD score, median (IQR)	973 (926–1032)	1000 (940–1064)	934 (924–981)	930 (905–936)
Other medical conditions				
Atrial fibrillation/flutter	39 763 (25.7%)	27 280 (27.4%)	9944 (23.7%)	2539 (19.2%)
Ischaemic heart disease	31 949 (20.6%)	21 368 (21.4%)	8381 (20.0%)	2200 (16.7%)
Prior percutaneous coronary interventions/coronary artery bypass graft	10 921 (7.1%)	7468 (7.5%)	2718 (6.5%)	735 (5.6%)
Peripheral vascular disease	6880 (4.4%)	4779 (4.8%)	1707 (4.1%)	394 (3.0%)
Prior valve replacement	2414 (1.6%)	1588 (1.6%)	657 (1.6%)	169 (1.3%)
Stroke	1030 (0.7%)	673 (0.7%)	278 (0.7%)	79 (0.6%)
Chronic pulmonary disease	21 769 (14.1%)	13 368 (13.4%)	6318 (15.1%)	2083 (15.8%)
Chronic kidney disease	17 480 (11.3%)	11 999 (12.0%)	4390 (10.5%)	1091 (8.3%)
Neurodegenerative disease^†^	7310 (4.7%)	4950 (5.0%)	1857 (4.4%)	503 (3.8%)
Malignancy	3301 (2.1%)	2113 (2.1%)	943 (2.2%)	245 (1.9%)
Cardiac risk factors				
Diabetes	42 435 (27.4%)	29 013 (29.1%)	10 279 (24.5%)	3143 (23.8%)
Hypertension	41 146 (26.6%)	28 783 (28.9%)	9561 (22.8%)	2802 (21.2%)
Hyperlipidaemia	8412 (5.4%)	6837 (6.9%)	1259 (3.0%)	316 (2.4%)
Charlson comorbidity index,^‡^ median (IQR)	1.0 (0–2)	1.0 (0–2)	1.0 (0–2)	0.0 (0–1)
Hospital length of stay (days),^§^ median (IQR)	5.0 (2–8)	5 (2–9)	4 (2–8)	4 (2–8)
Year group				
2002–2005	31 124	19 820 (63.7%)	8329 (26.8%)	2975 (9.6%)
2006–2009	28 345	17 970 (63.4%)	7814 (27.6%)	2561 (9.0%)
2010–2013	30 665	19 859 (64.8%)	8205 (26.8%)	2601 (8.5%)
2014–2017	33 028	21 513 (65.1%)	8923 (27.0%)	2592 (7.8%)
2018–2021	31 691	20 525 (64.8%)	8682 (27.4%)	2484 (7.8%)

IQR = interquartile range; IRSAD = Index of Relative Social Advantage and Disadvantage; SD = standard deviation.

* First admissions during the study period only.

† Includes dementia, central nervous systemic atrophies, Parkinson disease, basal ganglia degeneration, nervous systemic degenerative diseases.

‡ Includes myocardial infarction, heart failure, peripheral vascular disease, stroke, dementia, chronic pulmonary disease, connective tissue disease, peptic ulcer disease, liver disease (mild or moderate to severe), diabetes (with or without organ damage), hemiplegia, moderate to severe renal disease, any tumour (within past five years), lymphoma, leukaemia, metastatic solid tumour, acquired immunodeficiency syndrome.

§ Length of stay by 4‐year admission period is reported in the Supporting Information, table 3.

### In‐hospital mortality and hospital length of stay

During 2002–21, 9621 people admitted to hospital with heart failure (6.2%) died during their admissions; the proportion was 8.0% in 2002, and 4.9% in 2021 (Supporting Information, figure 2). By remoteness category, 5887 patients from metropolitan areas (5.9%), 2740 from inner regional areas (6.5%), and 994 people from outer regional/remote/very remote areas (7.5%) died in hospital (Box 2). By sex, 5005 women (6.6%) and 4615 men (5.9%) had died in hospital; the proportion of women who died in hospital exceeded that of men in all three remoteness categories: metropolitan, 3006 women (6.1%) and 2880 men (5.8%); inner regional, 1449 women (7.1%) and 1291 men (6.0%); outer regional/remote/very remote, 550 women (8.7%) and 444 men (6.4%) (Supporting Information, figure 3). In the multivariable analysis, the association of sex with in‐hospital mortality was not statistically significant (aOR, 0.98, 95% CI, 0.94–1.02) (Box 3).

Box 2 **Proportions of people aged 16 years or older who died during admissions to New South Wales public hospitals, 1 January 2002 – 30 September 2021, by remoteness of residence**

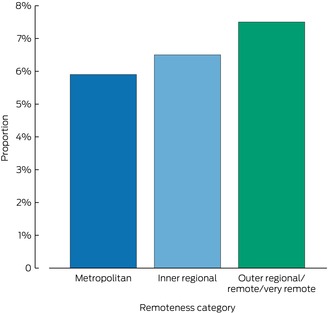

Box 3 **In‐hospital mortality during admissions with heart failure of people aged 16 years or older to New South Wales public hospitals, 1 January 2002 – 30 September 2021: logistic regression analysis**

**Table:** 

Parameter	Deaths	Missing cases	Univariable odds ratio (95% CI)	Multivariable odds ratio (95% CI)
Remoteness category				
Metropolitan	5887 (5.9%)	0	1	1
Inner regional	2740 (6.5%)	0	1.11 (1.06–1.17)	1.12 (1.07–1.17)
Outer regional/remote/very remote	994 (7.5%)	0	1.30 (1.21–1.39)	1.35 (1.25–1.45)
Age (years)*				
Older than 80.3 years old	6672 (8.6%)	0	2.32 (2.28–2.49)	2.21 (2.11–2.31)
80.3 years or younger	2949 (3.8%)	0	1	1
Sex				
Men	4615 (5.9%)	0	0.89 (0.85–0.93)	0.98 (0.94–1.02)
Women	5005 (6.6%)	0	1	1
IRSAD score*				
More than 973	4690 (6.1%)	0	0.96 (0.81–0.99)	0.96 (0.91–1.00)
973 or less	4931 (6.4%)	25	1	1
Ischaemic heart disease				
Yes	3131 (9.8%)	0	1.95 (1.86–2.04)	1.90 (1.81–1.98)
No	6490 (5.3%)	0	1	1
Prior percutaneous coronary interventions/coronary artery bypass graft				
Yes	514 (4.7%)	0	0.73 (0.67–0.80)	0.60 (0.54–0.65)
No	9107 (6.3%)	0	1	1
Stroke				
Yes	265 (25.7%)	0	5.35 (4.64–6.16)	3.47 (3.07–3.92)
No	9356 (6.1%)	0	1	1
Peripheral vascular disease				
Yes	666 (9.7%)	0	1.66 (1.53–1.81)	1.53 (1.41–1.66)
No	8955 (6.1%)	0	1	1
Atrial fibrillation/flutter				
Yes	2800 (7.0%)	0	1.20 (1.15–1.26)	1.05 (1.01–1.10)
No	6821 (5.9%)	0	1	1
Prior valve replacement				
Yes	148 (6.1%)	0	0.99 (0.83–1.17)	—
No	9473 (6.2%)	0	1	—
Chronic pulmonary disease				
Yes	1839 (8.4%)	0	1.49 (1.41–1.57)	1.44 (1.37–1.52)
No	7782 (5.8%)	0	1	1
Chronic kidney disease				
Yes	1637 (9.4%)	0	1.68 (1.58–1.77)	1.97 (1.86–2.08)
No	7894 (5.8%)	0	1	1
Malignancy				
Yes	581 (17.6%)	0	3.37 (3.07–3.69)	3.06 (2.81–3.33)
No	9040 (6.0%)	0	1	1
Neurodegenerative disease^†^				
Yes	1051 (14.4%)	0	2.73 (2.54–2.92)	1.85 (1.73–1.97)
No	8570 (5.8%)	0	1	1
Hypertension				
Yes	2506 (6.1%)	0	0.97 (0.93–1.02)	—
No	7115 (6.3%)	0	1	—
Hyperlipidaemia				
Yes	412 (4.9%)	0	0.77 (0.69–0.85)	0.66 (0.60–0.73)
No	9209 (6.3%)	0	1	1
Diabetes				
Yes	2223 (5.2%)	0	0.79 (0.75–0.82)	0.85 (0.81–0.89)
No	7398 (6.6%)	0	1	1
Hospital length of stay, per day	9621 (6.2%)	0	1.01 (1.01–1.01)	1.00 (1.00–1.00)
Year groups				
2002–2005	2584 (8.3%)	0	1	1
2006–2009	2209 (7.8%)	0	0.93 (0.88–0.99)	0.96 (0.90–1.01)
2010–2013	1852 (6.0%)	0	0.71 (0.67–0.76)	0.72 (0.68–0.77)
2014–2017	1631 (4.9%)	0	0.57 (0.54–0.61)	0.60 (0.56–0.64)
2018–2021	1345 (4.2%)	0	0.49 (0.46–0.52)	0.52 (0.49–0.56)

CI = confidence interval; IRSAD = Index of Relative Social Advantage and Disadvantage.

* Categories based on median values for all patients.

† Includes dementia, central nervous systemic atrophies, Parkinson disease, basal ganglia degeneration, nervous systemic degenerative diseases.

The median hospital length of stay was five days (IQR, 2–8 days); it was five days (IQR, 2–9 days) for patients from metropolitan areas, and four days (IQR, 2–8 days) for patients from both inner regional and outer regional/remote/very remote areas (Box 1).

### Change in in‐hospital all‐cause mortality during admissions with heart failure

In‐hospital all‐cause mortality was lower during 2018–21 than during 2002–2005, both overall (aOR, 0.52; 95% CI, 0.49–0.56) (Box 3; Box 4) and for patients from metropolitan areas (aOR, 0.50; 95% CI, 0.46–0.55), inner regional areas (aOR, 0.53; 95% CI, 0.47–0.61), and outer regional/remote/very remote areas (aOR, 0.55; 95% CI, 0.44–0.69) (Supporting Information, figure 4).

Box 4 **In‐hospital mortality during admissions with heart failure of people aged 16 years or older to New South Wales public hospitals, 1 January 2002 – 30 September 2021, by year group**

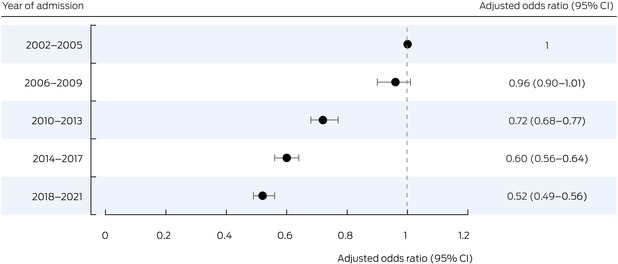

CI = confidence interval.

### Associations of patient factors with in‐hospital mortality

Compared with patients from metropolitan areas, the odds of in‐hospital death were higher for patients from inner regional (aOR, 1.12; 95% CI, 1.07–1.17) and outer regional/remote/very remote areas (aOR, 1.35; 95% CI, 1.25–1.45) (Box 5). Similar results were yielded by a multivariable model that additionally included CCI as a variable: inner regional: aOR, 1.15 (95% CI, 1.10–1.21); outer regional/remote/very remote areas: aOR, 1.37 (95% CI, 1.28–1.48) (Supporting Information, table 4). The odds of in‐hospital mortality were greater for people with a history of stroke (aOR, 3.47; 95% CI, 3.07–3.92) or malignancy (aOR, 3.06; 95% CI, 2.81–3.33) (Box 3).

Box 5 **In‐hospital mortality during admissions with heart failure of people aged 16 years or older to New South Wales public hospitals, 1 January 2002 – 30 September 2021, by remoteness category**

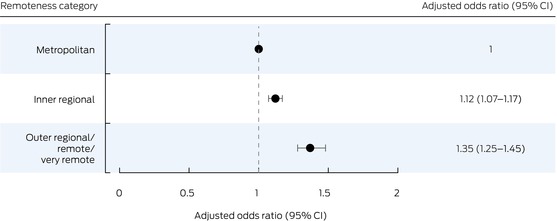

CI = confidence interval.

## Discussion

We found that all‐cause in‐hospital mortality among people admitted to NSW public hospitals with heart failure during 2002–21 declined significantly during the study period, but that it was higher among patients from regional or remote areas than people from metropolitan areas.

A 2018 meta‐analysis found few studies that had examined differences in outcomes of hospitalisations with heart failure for people from metropolitan and regional areas.^24^ We report the largest published study of in‐hospital mortality among people admitted to hospital with heart failure in Australia, and our 20‐year study period facilitated a comprehensive analysis of statewide changes in admissions and outcomes for a geographically diverse population.

We found that overall in‐hospital mortality for people hospitalised with heart failure during 2002–21 was 6.2%, but ranged from 5.9% for metropolitan area residents to 7.5% for people from outer regional/remote/very remote areas. The NSW Heart Failure Snapshot study, a four‐week audit of heart failure admissions to 24 participating NSW and Australian Capital Territory hospitals in mid‐2013, found that in‐hospital mortality was 6%;^25^ the authors did not state how many patients were from metropolitan and regional areas. A retrospective study found that in‐hospital mortality was 10.5% among people admitted with heart failure to hospitals in the Hunter New England Local Health District during 2005–2014, 40% of whom had been admitted to regional or remote hospitals.^26^ This earlier study was not statewide and was undertaken prior to recent therapeutic advances, but it was closer in design to our study than the NSW Heart Failure Snapshot study. Other Australian studies have examined 30‐day mortality for people from metropolitan and regional areas admitted to hospital with heart failure. In a 2014 Western Australian study, adjusted 30‐day mortality was higher among people from regional areas than those from metropolitan areas (OR, 1.16, 95% CI, 1.01–1.33);^11^ a 2022 Victorian study did not find any difference in 30‐day mortality, but fewer than 100 of the 1357 included patients were from regional areas.^27^

Our study was unique in that we assessed in detail temporal changes in in‐hospital mortality among people admitted with heart failure over twenty years. No other Australian study has examined statewide heart failure outcomes for such a long period. We found that all‐cause in‐hospital mortality was 48% lower for people admitted during 2018–21 than for those admitted during 2002–2005; the reduction was similar for people from metropolitan, inner regional, and outer regional/remote/very remote areas. Several factors are likely to have contributed to this change. Guideline‐directed medical therapies are important for minimising in‐hospital mortality among patients admitted with acute heart failure,^28^, ^29^ and non‐pharmacological measures, such as appropriate heart failure care pathways, also improve survival.^30^ We had no information about in‐hospital therapy, but the reduction in in‐hospital mortality could be partially attributable to improved pharmacological and non‐pharmacological management of heart failure in both metropolitan and regional areas.

We found that the likelihood of dying in hospital was higher for patients with heart failure from non‐metropolitan areas: 12% higher for inner regional residents, and 35% higher for people in outer regional/remote/very remote areas after adjusting for baseline characteristics, year of admission, socio‐economic status, and other medical conditions. Clinical outcomes are likely to be more favourable for people with heart failure if they receive care from a cardiologist rather than a general physician or general practitioner.^5^, ^6^ In the NSW Heart Failure Snapshot study, 8% of patients were in the care of heart failure physicians, 62% in the care of cardiologists, and 40% in the care of general physicians.^25^ A 2014 study found that general physicians or practitioners were more likely to provide inpatient heart failure care for people from regional areas than those from metropolitan Western Australia,^11^ and similar results were found by a study in Victoria.^27^ We did not have information about whether patients received specialist cardiology care at the 242 admitting hospitals in our study; 81 934 admissions (52.9%) were to twenty tertiary or quaternary hospitals and 15 017 (9.7%) to seventeen metropolitan district hospitals; 27 899 people (18.0%) were admitted to fifteen large regional hospitals and 30 003 (19.4%) to smaller district‐level or lower tier hospitals in outer regional/remote/very remote areas, which presumably did not have access to the cardiology services available in larger centres. More comprehensive information about admitting physicians at each institution would be needed to assess the influence of care type on outcomes for patients with heart failure from outside metropolitan areas.

As an analysis of population‐level data for the most populous Australian state, our findings provide a useful benchmark for in‐hospital mortality during admissions with heart failure. It could support assessment of the impact of inpatient heart failure care programs in regional Australia. Linking patient‐level admission and discharge medications information, outpatient follow‐up via Medicare Benefits Schedule billing codes, and individual echocardiographic data would facilitate a more comprehensive investigation of why in‐hospital mortality is higher for people from regional and remote areas with heart failure than for metropolitan patients.

### Limitations

The findings of our retrospective analysis of observational data can be used to generate hypotheses and could assist prospective studies of heart failure outcomes for people living in regional and remote areas. We did not apply a look‐back strategy to identify lifetime index heart failure admissions. It is likely that our reference group of people admitted during 2002–2005 included patients who had previously been admitted to hospital with heart failure; people with recurrent heart failure admissions are at greater risk of in‐hospital death. The continuous decline in‐hospital mortality during the study period suggests that this factor is unlikely to fully explain the improvement in survival at the end of the study period. The APDC does not include information about ejection fraction, medical therapy, haemodynamic profile, or blood pathology findings for individual patients.

Indigenous people have poorer heart failure outcomes than non‐Indigenous Australians,^24^, ^31^ and a larger proportion live in regional and remote areas.^3^ In‐hospital heart failure mortality among Australia Indigenous patients have not been published. The larger proportion of Indigenous patients in regional and remote areas may have influenced our findings, but the APDC does not include information about ethnic background.

The findings of our analysis of statewide data for the most populous Australian state should be generalisable to the entire country, but analysis of national data would be preferable. We limited our study to only people admitted with heart failure to public hospitals, excluding 13% of all heart failure admissions during the study period. Finally, we examined only in‐hospital death as an outcome for people admitted with heart failure, not the full spectrum of heart failure presentations, nor outcomes after leaving hospital.

### Conclusion

In‐hospital mortality is higher among people living in inner regional and outer regional/remote/very remote areas of NSW admitted to public hospitals with heart failure than for people living in metropolitan areas. Detailed patient‐level information is needed to identify the reasons for this difference, enabling targeted programs for improving outcomes for people with heart failure in regional and remote Australia.

## Open access

Open access publishing facilitated by the University of Sydney, as part of the Wiley – the University of Sydney agreement via the Council of Australian University Librarians.

## Competing interests

No relevant disclosures.

## Data sharing

The NSW Population and Health Services Research Ethics Committee (PHSREC) prohibits authors from making the minimal data set publicly available. Interested researchers may contact the ethics coordinator (ethics@cancerinstitute.org.au) to seek permission to access the data; data will then be made available upon request to interested researchers who receive approval from the NSW PHSREC.

## Supporting information


Supplementary methods and results
