# Supplementary material for: Geographic remoteness‐based differences in in‐hospital mortality among people admitted to NSW public hospitals with heart failure, 2002–21: a retrospective observational cohort study
Source: Med J Aust. 2025 Apr 20;222(7):348–55. doi: 10.5694/mja2.52635 (PMC12009594; doi:10.5694/mja2.52635)
Supplement: Supplementary file 1 — Supplementary methods and results [file MJA2-222-348-s001.pdf]

The Medical Journal of Australia

Supporting Information

Supplementary methods and results

This appendix was part of the submitted manuscript and has been peer reviewed.
It is posted as supplied by the authors.

Appendix to: Rubenis I, Harvey G, Hyun K, et al. Geographic remoteness-based differences in in-hospital mortality among people admitted to NSW public hospitals with heart failure, 2002–21: a retrospective observational cohort study. *Med J Aust* 2025; doi: 10.5694/mja2.52635.

Supplementary methods

Table 1. International Statistical Classification of Diseases and Related Health Problems, Tenth Revision, Australian Modification (ICD-10-AM) codes for medical conditions included in analysis

No.	Medical condition*	ICD-10-AM codes
1	Atrial fibrillation/flutter	I48, I48.0, I48.1, I48.2, I48.3, I48.4, I48.9
2	Acute myocardial infarction	I21, I21.0, I21.1, I21.2, I21.3, I21.4, I21.9, I22, I22.0, I22.1, I22.8, I22.9
3	Ischemic heart disease	I20, I20.0, I20.1, I20.8, I20.9, I21, I21.0, I21.1, I21.2, I21.3, I21.4, I21.9, I22, I22.0, I22.1, I22.8, I22.9, I23, I23.0, I23.1, I23.2, I23.3, I23.4, I23.5, I23.6, I23.8, I24, I24.0, I24.1, I24.8, I24.9, I25, I25.0, I25.1, I25.10, I25.11, I25.12, I25.13, I25.2, I25.3, I25.4, I25.5, I25.6, I25.8, I25.9
4	Prior percutaneous coronary intervention (PCI) / coronary artery bypass grafting (CABG)	Z95.1, Z95.5
5	Heart failure	I42, I42.0, I42.1, I42.2, I42.3, I42.4, I42.5, I42.6, I42.7, I42.8, I42.9, I43, I43.0, I43.1, I43.2, I43.8, I50, I50.0, I50.1, I50.9, I11.0, I13.0, I13.2
6	Peripheral vascular disease	E09.5, E09.51, E09.52, E10.51, E10.52, E11.51, E11.52, E13.51, E13.52, E14.51, E14.52, I70, I70.0, I70.1, I70.2, I70.20, I70.21, I70.22, I70.23, I70.24, I70.8, I70.9, I71, I71.0, I71.00, I71.01, I71.02, I71.03, I71.1, I71.2, I71.3, I71.4, I71.5, I71.6, I71.8, I71.9, I72, I72.0 I72.1, I72.2, I72.3, I72.4, I72.5, I72.6, I72.8, I72.9, I73, I73.0, I73.1, I73.8, I73.9, I74, I74.0, I74.1, I74.2, I74.3, I74.4, I74.5, I74.8, I74.9, I77, I77.0, I77.1, I77.2, I77.3, I77.4, I77.5, I77.6, I77.8, I77.9, I78, I78.0, I78.1, I78.8, I78.9, I79, I79.0, I79.1, I79.2, I79.8
7	Stroke	G45, G45.0, G45.1, G45.2, G45.3, G45.4, G45.8, G45.9, G46, G46.0, G46.1, G46.2, G46.3, G46.4, G46.5, G46.6, G46.7, G46.8, I60, I60.0, I60.1, I60.2, I60.3, I60.4, I60.5, I60.6, I60.7, I60.8, I60.9, I61, I61.0, I61.1, I61.2, I61.3, I61.4, I61.5, I61.6, I61.8, I61.9, I62, I62.0, I62.1, I62.9, I63, I63.0, I63.1, I63.2, I63.3, I63.4, I63.5, I63.6, I63.8, I63.9, I64
8	Prior valve replacement	Z95.2, Z95.3, Z95.4
9	Hypertension	I10, I11, I11.0, I11.9, I12, I12.0, I12.9, I13, I13.0, I13.1, I13.2, I13.9, I15, I15.0, I15.1, I15.2, I15.8, I15.9
10	Hyperlipidemia	E78, E78.0, E78.1, E78.2, E78.3, E78.4, E78.5, E78.6, E78.8, E78.9
11	Diabetes	E09, E09.2, E09.21, E09.29, E09.3, E09.31, E09.32, E09.4, E09.40, E09.42, E09.5, E09.51, E09.52, E09.7, E09.71, E09.72, E09.8, E09.9, E10, E10.0, E10.01, E10.02, E10.1, E10.11, E10.12, E10.13, E10.14, E10.15, E10.16, E10.2, E10.21, E10.22, E10.29, E10.3, E10.31, E10.32, E10.33, E10.34, E10.35, E10.36, E10.39, E10.4, E10.40, E10.41, E10.42, E10.43, E10.49, E10.5, E10.51, E10.52, E10.53, E10.6, E10.61, E10.62, E10.63, E10.64, E10.65, E10.69, E10.7, E10.71, E10.73, E10.8, E10.9, E11, E11.0, E11.01, E11.02, E11.1, E11.11, E11.12, E11.13, E11.14, E11.15, E11.16, E11.2, E11.21, E11.22, E11.29, E11.3, E11.31, E11.32, E11.33, E11.34, E11.35, E11.36, E11.39, E11.4, E11.40, E11.41, E11.42, E11.43, E11.49, E11.5, E11.51, E11.52, E11.53, E11.6, E11.61, E11.62, E11.63, E11.64, E11.65, E11.69, E11.7, E11.71, E11.72, E11.73, E11.8, E11.9, E13, E13.0, E13.01, E13.02, E13.1, E13.11, E13.12, E13.13, E13.14, E13.15, E13.16, E13.2, E13.21, E13.22, E13.29, E13.3, E13.31, E13.32, E13.33, E13.34, E13.35, E13.36, E13.39, E13.4, E13.40, E13.41, E13.42, E13.43, E13.49, E13.5, E13.51, E13.52, E13.53, E13.6, E13.61, E13.62, E13.63, E13.64, E13.65, E13.69, E13.7, E13.71, E13.72, E13.73, E13.8, E13.9, E14, E14.0, E14.01, E14.02, E14.1, E14.11, E14.12, E14.13, E14.14, E14.15, E14.16,

No.	Medical condition*	ICD-10-AM codes
		E14.2, E14.21, E14.22, E14.29, E14.3, E14.31, E14.32, E14.33, E14.34, E14.35, E14.36, E14.39, E14.4, E14.40, E14.41, E14.42, E14.43, E14.49, E14.5, E14.51, E14.52, E14.53, E14.6, E14.61, E14.62, E14.63, E14.64, E14.65, E14.69, E14.7, E14.71, E14.72, E14.73, E14.8, E14.9, Z92.22
12	Current/ex-smoker	F17, Z72.0, Z86.43
13	Systemic connective tissue disease	M30, M30.0, M30.1, M30.2, M30.3, M30.8, M31, M31.0, M31.1, M31.2, M31.3, M31.4, M31.5, M31.6, M31.7, M31.8, M31.9, M32, M32.0, M32.1, M32.8, M32.9, M33, M33.0, M33.1, M33.2. M33.9, M34, M34.0, M34.1, M34.2, M34.8, M34.9, M35, M35.0, M35.1, M35.2, M35.3, M35.4, M35.5, M35.6, M35.7, M35.8, M35.9, M36, M36.0, M36.1, M36.2, M36.3, M36.4, M36.8
14	Chronic pulmonary disease<br>(include asthma, chronic airways limitation, interstitial lung disease, cystic fibrosis with pulmonary manifestation)	E84.0, E84.0, E84.1, E84.8, E84.9, J40, J41, J41.0, J41.1, J41.8, J42, J43, J43.0, J43.1, J43.2, J43.8, J43.9, J44, J44.0, J44.1, J44.8, J44.9, J45, J45.0, J45.1, J45.8, J45.9, J46, J47, J60, J61, J62, J62.0, J62.8, J63, J63.0, J63.1, J63.2, J63.3, J63.4, J63.5, J63.8, J64, J65, J66, J66.0, J66.1, J66.2, J66.8, J67, J67.0, J67.1, J67.2, J67.3, J67.4, J67.5, J67.6, J67.7, J67.8, J67.9, J68, J68.0, J68.1, J68.2, J68.3, J68.4, J68.8, J68.9, J70, J70.0, J70.1, J70.2, J70.3, J70.4, J70.8, J70.9, J82, J84, J84.0, J84.1, J84.8. J84.9, J99, J99.1, J99.8
15	Malignancy	C00, C00.0, C00.1, C00.2, C00.3, C004, C00.5, C00.6, C00.8, C00.9, C01, C02, C02.0, C02.1, C02.2, C02.3, C02.4, C02.8, C02.9,C03, C03.0, C03.1 C03.9, C04, C04.0, C04.1, C04.8, C04.9, C06.0, C06.1, C06.2, C06.8, C06.9, C05, C05.0, C05.1, C05.2, C05.8, C05.9, D04.0, D00.0, D04.1, D04.2, D04.3, D04.4, C07, C08, C08.0, C08.1, C08.8, C08.9, C09, C09.0, C09.1, C09.8, C09.9, C10, C10.0, C10.1, C10.2, C10.3, C10.4, C10.8, C10.9, C14.2, C14.0, C11, C11.0, C11.1, C11.2, C11.3, C11.8, C11.9, C12, C13, C13.0, C 13.1, C13.2, C13.8, C13.9, C14, C14.0, C14.2, C14.8, C30, C30.0, C30.1, C31, C31.1, C31.2, C31.3, C31.8, C31.9, C32, C32.1, C32.2, C32.3, C32.8, C32.9, C33, D02.0, D02.1, D02.3, C17, C17.0, C17.1, C 17.2, C17.3, C17.8, C17.9, C18, C18.0, C18.1, C18.2, C18.3, C18.4, C18.5, C18.6, C18.7, C18.8, C18.9, C19, C26.0, C26.8, C26.9, C20, C21, C21.0, C21.1, C21.2, C21.8, D01.0, D01.1, D01.2, D01.3, D01.4, C22, C22.0, C22.1, C22.2, C22.3, C22.4, C22.7, C22.9, C23, C24, C24.0, C24.1, C24.8, C24.9, C25, C25.0, C25.1, C25.2, C25.3, C25.4, C25.7, C25.8, C25.9, D01.5, D01.7, C15, C15.0, C15.1, C15.2, C15.3, C15.4, C15.5, C15.8, C15.9, C16 C16.0, C16.1, C16.2, C16.3, C16.4, C16.5, C16.6, C16.8, C16.9, C26.1, C26, D00.1, D00.2, D01.9, C34, C34.0, C34.1, C34.2, C34.3, C34.8, C34.9, C38.4, D02.2, D02.3, D02.4, C40, C40.0, C40.1, C40.2, C40.3, C40.8, C40.9, C41, C41.1, C41.2, C41.3, C41.4, C41.8, C41.9, C49, C49.0, C49.1, C49.2, C49.3, C49.4, C49.5, C49.6, C49.8, C49.9, C50, C50.0, C50.1, C50.2, C50.3, C50.4, C50.5, C50.6, C50.8, C50.9, D05, D05.0, D05.1, D05.7, D05.9, C53, C53.0, C53.1, C53.8, C53.9, D06, D06.0, D06.1, D06.7, D06.9, C54, C54.0, C54.1, C54.2, C54.3, C54.8, C54.9, C55, D07.0, D07.2, D07.3, C56, C57, C57.0, C57.1, C57.2, C57.3, C57.4, C57.7, C57.8, C57.9, C58, C51, C51.0, C51.1, C51.2, C51.8, C51.9, C52<br>C43, C43.0, C43.1, C43.2, C43.3, C43.4, C43.5, C43.6, C43.7, C43.8, C43.9, D03, D03.0, D03.1, D03.2, D03.3, D03.4, D03.5, D03.6, D03.7, D03.8, D03.9, C44, C44.0, C44.1, C44.2, C44.3, C44.4, C44.5, C44.6, C44.7, C44.8, C44.9, C63.2, D04.5, D04.6, D04.7, D04.8, D04.9, C61, D07.5, C62, C62.0, C62.1, C62.9, C63, C63.0, C63.1, C63.7, C63.8, C63.9, D07.6, C60, C60.0, C60.1, C60.2, C60.8, C60.9, D07.4, C64, C65, C66, C67, C67.0, C67.1, C67.2, C67.3, C67.4, C67.5, C67.6, C67.7, C67.8, C67.9,

No.	Medical condition*	ICD-10-AM codes
		D09.0, C68, C68.0, C68.1, C68.8, C68.9, D09.1, C80, C80.0. C80.9, D09.7, D09.9, C97, C76, C76.0, C76.1, C76.2, C76.3, C76.4, C76.5, C76.8, C69, C69.0, C69.1, C69.2, C69.3, C69.4, C69.5, C69.6, C69.8, C69.9, D09.2, C70, C70.0, C70.1, C70.9, C71, C71.0, C71.1, C71.2, C71.3, C71.4, C71.5, C71.6, C71.7, C71.8, C71.9, C72.8, C72, C72.1, C72.2, C72.3, C72.4, C72.5, C72.9, C37.1, C38.2, C38.3, C37, C38, C38.0, C38.8, C39, C39.0, C39.8, C39.9, C45, C45.0, C45.1, C45.2, C45.7, C45.9, C46, C46.0, C46.1, C46.2, C46.3, C46.7, C46.8, C46.9, C47, C47.0, C47.1, C47.2, C47.3, C47.4, C47.5, C47.6, C47.8, C47.9, C48, C48.1, C48.2, C48.8, C73, D09.3, C74, C74.0, C74.1, C74.9, C75, C75.0, C75.1, C75.2, C75.3, C75.4, C75.5, C75.8, C75.9, C77, C77.0, C77.1, C77.2, C77.3, C77.4, C77.5, C77.8, C77.9, C78.0, C78.1, C78.2, C78.3, C78.4, C78.5, C78.6, C78.7, C78.8, C79.0, C79.1, C79.2, C79.3, C79.4, C79.5, C79.6, C79.7, C79.8, C79.9, C81, C81.0, C81.2, C81.3, C81.4, C81.7, C81.9, C82, C82.0, C82.1, C82.2, C82.3, C82.4, C82.5, C82.6, C82.7, C82.9, C83, C83.0, C83.1, C83.3, C83.5, C83.7, C83.8, C83.9, C84, C84.0, C84.1, C84.4, C84.5, C84.6, C84.7, C84.8, C84.9, C85, C85.1, C85.2, C85.7, C85.9, C86, C86.0, C86.1, C86.2, C86.3, C86.4, C86.5, C86.6, C90, C90.0, C90.1, C90.2, C90.3, C91, C91.0, C91.1, C91.3, C91.4, C91.5, C91.6, C91.7, C91.8, C91.9, C92, C92.0, C92.1, C92.2, C92.3, C92.4, C92.5, C92.6, C92.7, C92.8, C92.9, C93, C93.0, C93.1, C93.3, C93.7, C93.9, C94, C94.0, C94.2, C94.3, C94.4, C94.6, C94.7, C95, C95.0, C95.1, C95.7, C95.9, C96, C96.0, C96.2, C96.4, C96.5, C96.6, C96.7, C96.8, C96.9, C88, C88.0, C88.2, C88.3, C88.4, C88.7, C88.9
16	Chronic kidney disease	N18, N18.1, N18.2, N18.3, N18.4, N18.5, N18.9, N19
17	Dementia	F00, F00.0, F00.1, F00.2, F00.9, F01, F01.0, F01.1, F01.2, F01.3, F01.8, F01.9, F02, F02.0, F02.1, F02.2, F02.3, F02.4, F02.8, F03
18	Neurodegenerative diseases (dementia, central nervous systemic atrophies, Parkinson disease, basal ganglia degeneration, nervous systemic degenerative diseases)	F00, F00.0, F00.1, F00.2, F00.9, F01, F01.0, F01.1, F01.2, F01.3, F01.8, F01.9, F02, F02.0, F02.1, F02.2, F02.3, F02.4, F02.8, F03, G10, G11, G11.0, G11.1, G11.2, G11.3, G11.4, G11.8, G11.9, G12, G12.0, G12.1, G12.2, G12.8, G12.9, G13, G13.0, G13.1, G13.2, G13.8, G14, G20, G23, G23.0, G23.1, G23.2, G23.8, G23.9, G30, G30.0, G30.1, G30.8, G30.9, G31, G31.0, G31.1, G31.2, G31.3, G31.8, G31.9
19	Peptic ulcer disease	K25, K26, K27, K28
20	Liver disease – mild	K70.0, K70.1, K70.2, K70.9, K71.0, K71.1, K71.2, K71.3, K71.4, K71.5, K71.6, K71.8, K71.9, K73, K73.0, K73.1, K73.2, K73.8, K73.9, K75, K75.0, K75.1, K75.2, K75.3, K75.4, K75.8, K75.9, K76, K76.0, K76.1, K76.2, K76.3, K76.4, K76.5, K76.6, K76.7, K76.8, K76.9, K77, K77.0, K77.8
21	Liver disease – moderate-severe	I82.0, K70.3, K70.4, K71.7, K72, K72.0, K72.1, K72.9, K74, K74.0, K74.1, K74.2, K74.3, K74.4, K74.5, K74.6
22	Chronic kidney disease – moderate-severe	N18.3, N18.4, N18.5
23	Diabetes with organ damage	E09.21, E09.29, E09.31, E09.32, E09.40, E09.42, E09.51, E09.52, E09.71, E09.72, E09.8, E10.21, E10.22, E10.29, E10.31, E10.32, E10.33, E10.34, E10.35, E10.36, E10.39, E10.40, E10.41, E10.42, E10.43, E10.49, E10.51, E10.52, E10.53, E10.61, E10.62, E10.63, E10.69, E10.71, E10.73, E10.8, E11.21, E11.22, E11.29, E11.31, E11.32, E11.33, E11.34, E11.35, E11.36, E11.39, E11.40, E11.41, E11.42, E11.43, E11.49, E11.51, E11.52, E11.53, E11.61, E11.62, E11.63, E11.69, E11.71, E11.72, E11.73, E11.8, E13.21, E13.22, E13.29, E13.31, E13.32, E13.33, E13.34, E13.35, E13.36, E13.39, E13.40, E13.41, E13.42, E13.43, E13.49, E13.51, E13.52, E13.53, E13.61, E13.62, E13.63, E13.69,

No.	Medical condition*	ICD-10-AM codes
		E13.71, E13.72, E13.73, E13.8, E14.21, E14.22, E14.29, E14.31, E14.32, E14.33, E14.34, E14.35, E14.36, E14.39, E14.40, E14.41, E14.42, E14.43, E14.49, E14.51, E14.52, E14.53, E14.61, E14.62, E14.63, E14.69, E14.71, E14.72, E14.73, E14.8
24	Lymphoma	C81, C81.0, C81.1, C81.2, C81.3, C81.4, C81.7, C81.9, C82, C82.0, C82.1, C82.2, C82.3, C82.4, C82.5, C82.6, C82.7, C82.9, C83, C83.0, C83.1, C83.3, C83.5, C83.7, C83.8, C83.9, C84, C84.0, C84.1, C84.4, C84.5, C84.6, C84.7, C84.8, C84.9, C85, C85.1, C85.2, C85.7, C85.9, C86, C86.0, C86.1, C86.2, C86.3, C86.4, C86.5, C86.6, C88, C88.0, C88.2, C88.3, C88.4, C88.7, C88.9
25	Leukaemia	C90, C90.0, C90.1, C90.2, C90.3, C91, C91.0, C91.1, C91.3, C91.4, C91.5, C91.6, C91.7, C91.8, C91.9, C92, C92.0, C92.1, C92.2, C92.3, C92.4, C92.5, C92.6, C92.7, C92.8, C92.9, C93, C93.0, C93.1, C93.3, C93.7, C93.9, C94, C94.0, C94.2, C94.3, C94.4, C94.6, C94.7, C95, C95.0, C95.1, C95.7, C95.9, C96, C96.0, C96.2, C96.4, C96.5, C96.6, C96.7, C96.8, C96.9
26	Metastatic solid tumour	C76, C76.0, C76.1, C76.2, C76.3, C76.4, C76.5, C76.7, C76.8, C77, C77.0, C77.1, C77.2, C77.3, C77.4, C77.5, C77.8, C77.9, C78, C78.0, C78.1, C78.2, C78.3, C78.4, C78.5, C78.6, C78.7, C78.8, C79, C79.0, C79.1, C79.2, C79.3, C79.4, C79.5, C79.6, C79.7, C79.8, C79.81, C79.82, C79.88, C79.9, C80, C80.0, C80.9
27	Hemiplegia	G81, G81.0, G81.1, G81.9, G82, G82.0, G82.1, G82.2, G82.3, G82.4, G82.5
28	Acquired immune deficiency syndrome (AIDS)	B20, B21, B22, B23, B23.0, B23.8, B24
29	Any tumour/malignancy excluding lymphoma and leukemia	C00, C00.0, C00.1, C00.2, C00.3, C004, C00.5, C00.6, C00.8, C00.9, C01, C02, C02.0, C02.1, C02.2, C02.3, C02.4, C02.8, C02.9,C03, C03.0, C03.1 C03.9, C04, C04.0, C04.1, C04.8, C04.9, C06.0, C06.1, C06.2, C06.8, C06.9, C05, C05.0, C05.1, C05.2, C05.8, C05.9, C07, C08, C08.0, C08.1, C08.8, C08.9, C09, C09.0, C09.1, C09.8, C09.9, C10, C10.0, C10.1, C10.2, C10.3, C10.4, C10.8, C10.9, C14.2, C14.0, C11, C11.0, C11.1, C11.2, C11.3, C11.8, C11.9, C12, C13, C13.0, C 13.1, C13.2, C13.8, C13.9, C14, C14.0, C14.2, C14.8, C30, C30.0, C30.1, C31, C31.1, C31.2, C31.3, C31.8, C31.9, C32, C32.1, C32.2, C32.3, C32.8, C32.9, C33, C17, C17.0, C17.1, C 17.2, C17.3, C17.8, C17.9, C18, C18.0, C18.1, C18.2, C18.3, C18.4, C18.5, C18.6, C18.7, C18.8, C18.9, C19, C26.0, C26.8, C26.9, C20, C21, C21.0, C21.1, C21.2, C21.8, C22, C22.0, C22.1, C22.2, C22.3, C22.4, C22.7, C22.9, C23, C24, C24.0, C24.1, C24.8, C24.9, C25, C25.0, C25.1, C25.2, C25.3, C25.4, C25.7, C25.8, C25.9, C15, C15.0, C15.1, C15.2, C15.3, C15.4, C15.5, C15.8, C15.9, C16 C16.0, C16.1, C16.2, C16.3, C16.4, C16.5, C16.6, C16.8, C16.9, C26.1, C26, C34, C34.0, C34.1, C34.2, C34.3, C34.8, C34.9, C38.4, C40, C40.0, C40.1, C40.2, C40.3, C40.8, C40.9, C41, C41.1, C41.2, C41.3, C41.4, C41.8, C41.9, C49, C49.0, C49.1, C49.2, C49.3, C49.4, C49.5, C49.6, C49.8, C49.9, C50, C50.0, C50.1, C50.2, C50.3, C50.4, C50.5, C50.6, C50.8, C50.9, C53, C53.0, C53.1, C53.8, C53.9, C54, C54.0, C54.1, C54.2, C54.3, C54.8, C54.9, C55, C56, C57, C57.0, C57.1, C57.2, C57.3, C57.4, C57.7, C57.8, C57.9, C58, C51, C51.0, C51.1, C51.2, C51.8, C51.9, C52, C43, C43.0, C43.1, C43.2, C43.3, C43.4, C43.5, C43.6, C43.7, C43.8, C43.9, C44, C44.0, C44.1, C44.2, C44.3, C44.4, C44.5, C44.6, C44.7, C44.8, C44.9, C63.2, C61, C62, C62.0, C62.1, C62.9, C63, C63.0, C63.1, C63.7, C63.8, C63.9, C60, C60.0, C60.1, C60.2, C60.8, C60.9, C64, C65, C66, C67, C67.0, C67.1, C67.2, C67.3, C67.4, C67.5, C67.6, C67.7, C67.8, C67.9, C68, C68.0, C68.1, C68.8, C68.9, C80, C80.0. C80.9, C76, C76.0, C76.1, C76.2, C76.3, C76.4, C76.5, C76.8, C69,

No.	Medical condition*	ICD-10-AM codes
		C69.0, C69.1, C69.2, C69.3, C69.4, C69.5, C69.6, C69.8, C69.9, D09.2, C70, C70.0, C70.1, C70.9, C71, C71.0, C71.1, C71.2, C71.3, C71.4, C71.5, C71.6, C71.7, C71.8, C71.9, C72.8, C72, C72.1, C72.2, C72.3, C72.4, C72.5, C72.9, C37.1, C38.2, C38.3, C37, C38, C38.0, C38.8, C39, C39.0, C39.8, C39.9, C45, C45.0, C45.1, C45.2, C45.7, C45.9, C46, C46.0, C46.1, C46.2, C46.3, C46.7, C46.8, C46.9, C47, C47.0, C47.1, C47.2, C47.3, C47.4, C47.5, C47.6, C47.8, C47.9, C48, C48.1, C48.2, C48.8, C73, C74, C74.0, C74.1, C74.9, C75, C75.0, C75.1, C75.2, C75.3, C75.4, C75.5, C75.8, C75.9, C77, C77.0, C77.1, C77.2, C77.3, C77.4, C77.5, C77.8, C77.9, C78.0, C78.1, C78.2, C78.3, C78.4, C78.5, C78.6, C78.7, C78.8, C79.0, C79.1, C79.2, C79.3, C79.4, C79.5, C79.6, C79.7, C79.8, C79.9; D00.0, D00.1, D00.2, D01.9, D01.0, D01.1, D01.2, D01.3, D01.4, D01.5, D01.7, D02.0, D02.1, D02.2, D02.3, D02.4, D03, D03.0, D03.1, D03.2, D03.3, D03.4, D03.5, D03.6, D03.7, D03.8, D03.9, D04.0, D04.1, D04.2, D04.3, D04.4, D04.5, D04.6, D04.7, D04.8, D04.9, D05, D05.0, D05.1, D05.7, D05.9, D06, D06.0, D06.1, D06.7, D06.9, D07.0, D07.2, D07.3, D07.4, D07.5, D07.6, D09.0, D09.1, D09.3, D09.7, D09.9

* We calculated the Charlson comorbidity index (CCI) score, without age adjustment, for individuals during an admission of interest using the following condition item numbers and their corresponding ICD-10-AM codes:

1 point for each item: 2, 5, 6, 7, 11, 13, 14, 17, 19, 20;
2 points for each item – 22, 23, 24, 25, 27, 29;
3 points for item 21;
6 points for each item – 26, 28.

Supplementary results

Figure 1. Selection of cases for inclusion in our analysis

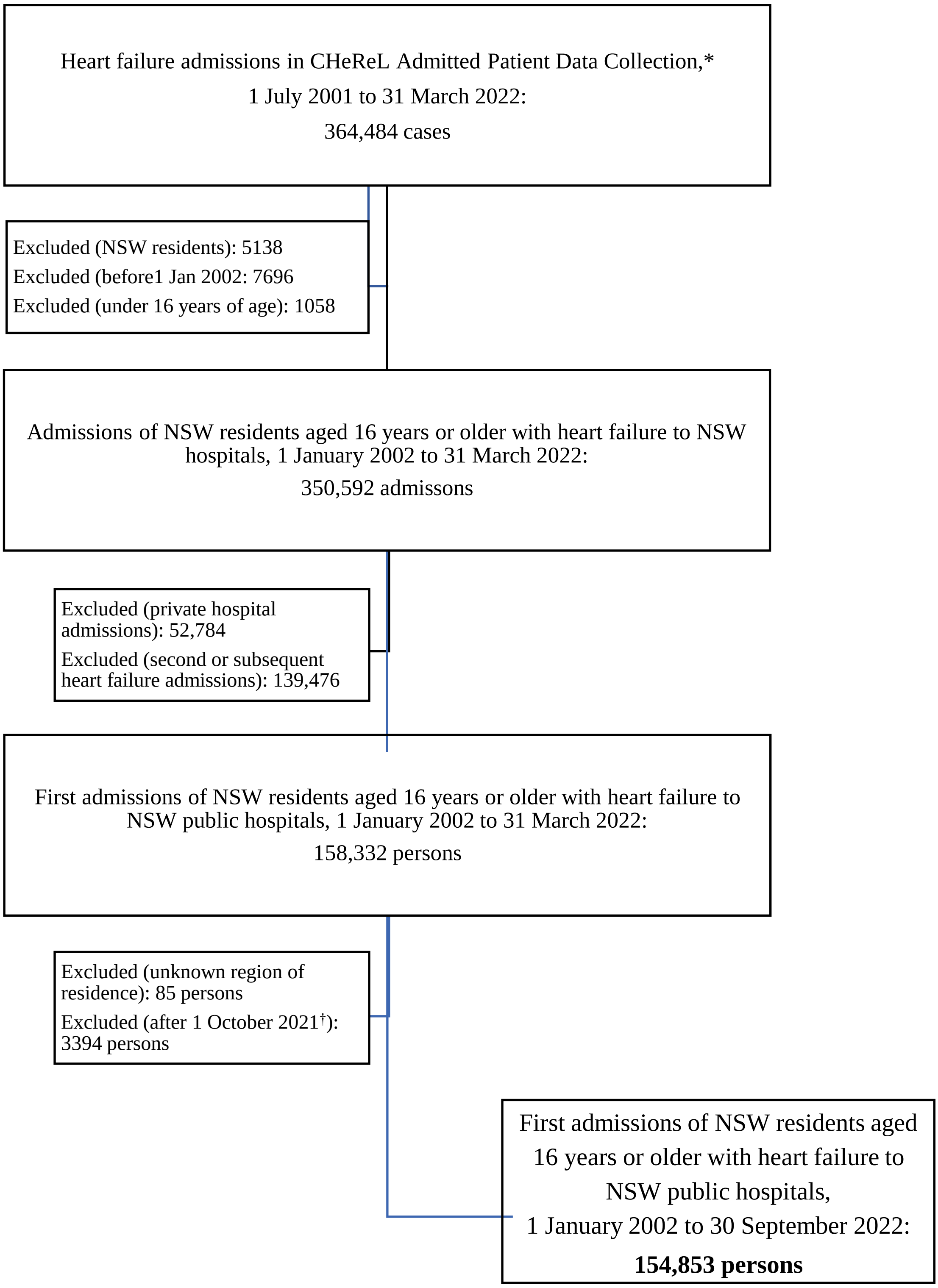

* Admissions with a primary diagnosis of heart failure: International Statistical Classification of Diseases, tenth revision, Australian modification (ICD-10-AM) codes I42, I43, I50, I11.0, I13.0, I13.2.

† The lag time for hospitals forwarding administrative data to CHeReL is about six months.

Table 3. Median length of hospital admission, by remoteness category

Year Group	Total	Metropolitan	Inner regional	Outer regional/remote
All years	5.0 (2 – 8)	5.0 (2 – 9)	4.0 (2 – 8)	4.0 (2 – 8)
2002 – 2005	5.0 (3 – 9)	5.0 (2 – 9)	5.0 (2 – 9)	5.0 (2 – 8)
2006 – 2009	5.0 (2 – 9)	5.0 (3 – 9)	5.0 (2 – 8)	4.0 (2 – 8)
2010 - 2013	5.0 (2 – 8)	4.0 (3 – 9)	4.0 (2 – 8)	4.0 (2 – 7)
2014 - 2017	4.0 (2 – 8)	4.0 (2 – 8)	4.0 (2 – 7)	4.0 (2 – 7)
2018 - 2021	4.0 (2 – 8)	5.0 (2 – 8)	4.0 (2 – 7)	4.0 (2 – 7)

Figure 2. Unadjusted in-hospital mortality, by remoteness category and year

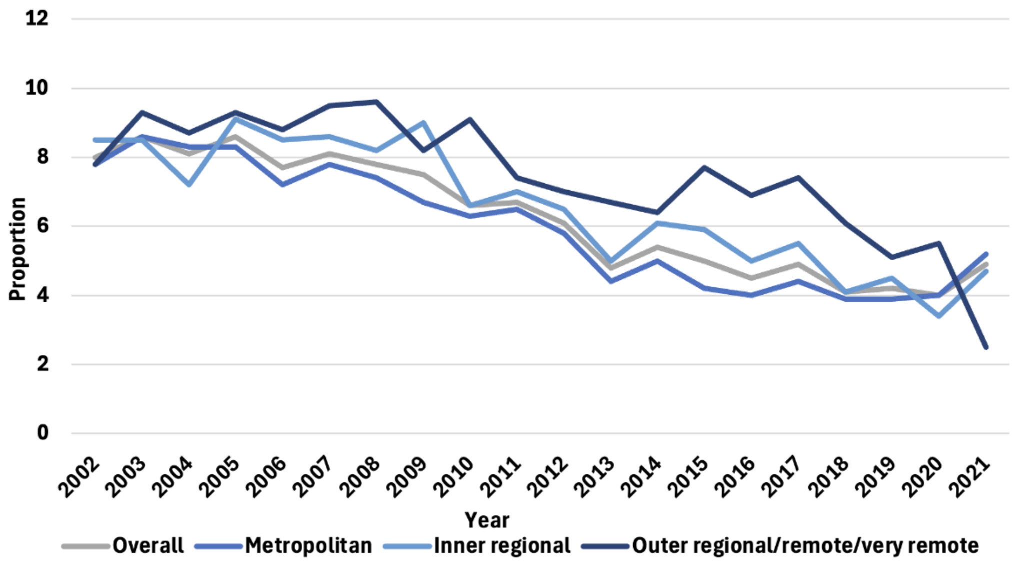

Figure 3. Unadjusted in-hospital heart failure mortality, by sex and remoteness category

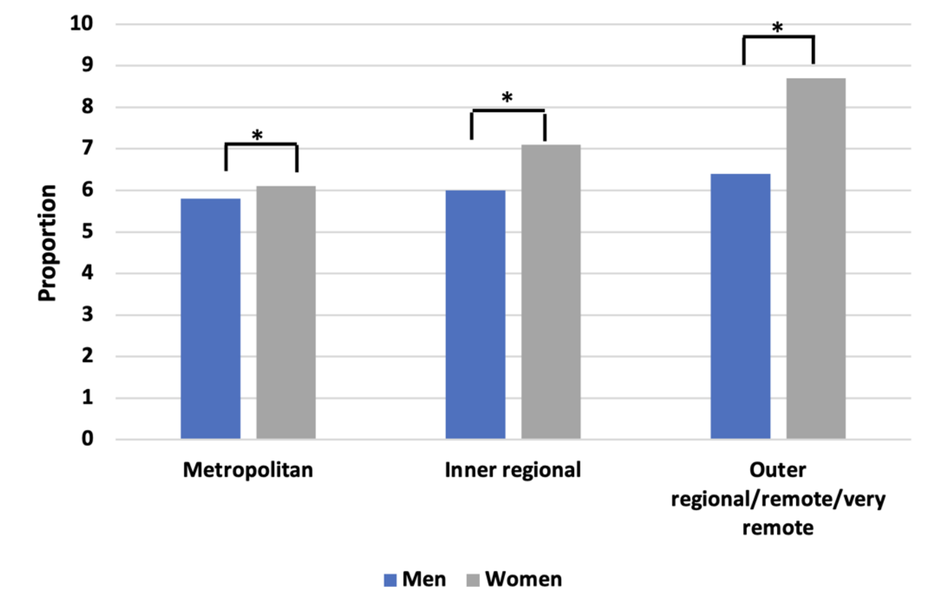

Figure 4. In-hospital mortality during admissions with heart failure of people aged 16 years or older to New South Wales public hospitals, 1 January 2002 – 30 September 2021, by year group and remoteness category

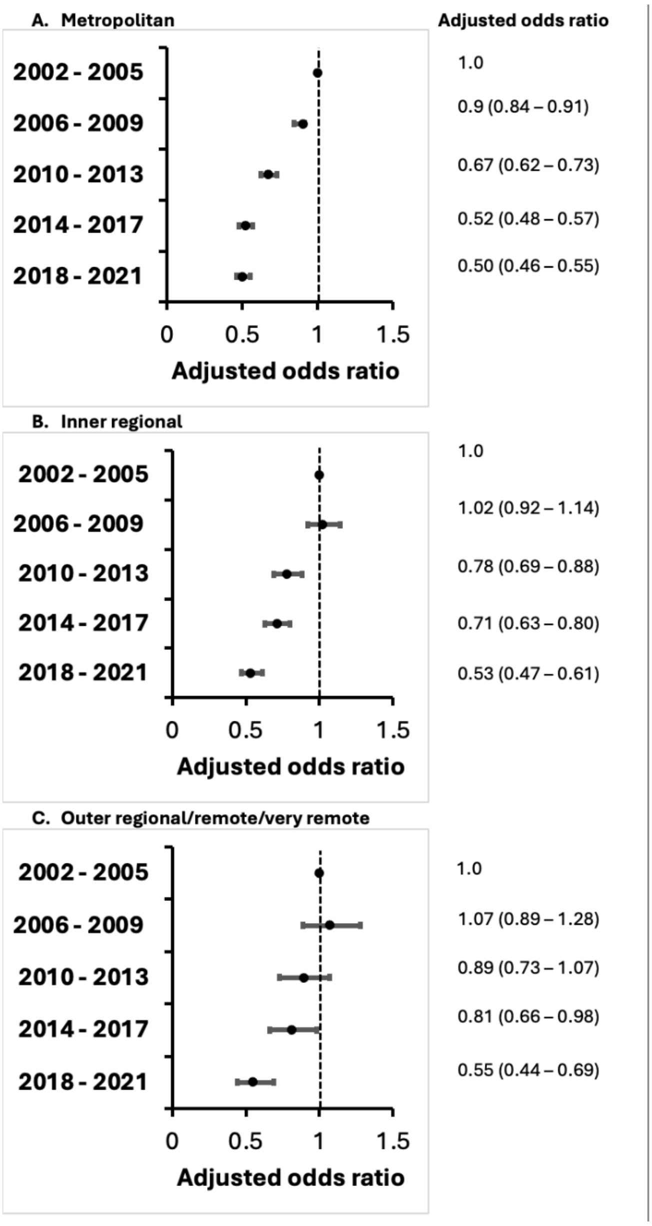

Table 4. In-hospital mortality during admissions with heart failure of people aged 16 years or older to New South Wales public hospitals, 1 January 2002 – 30 September 2021: logistic regression analysis including Charlson comorbidity index as variable

**Parameter**	**Univariable odds ratio (95% CI)**	**Multivariable odds ratio (95% CI)***
Remoteness category		
Metropolitan	1	1
Inner regional	1.11 (1.06–1.17)	1.15 (1.10–1.21)
Outer regional/remote/very remote	1.30 (1.21–1.39)	1.37 (1.28–1.48)
Age (older than median)	2.32 (2.28–2.49)	2.53 (2.42–2.65)
Sex (men)	0.89 (0.85–0.93)	0.99 (0.95–1.03)
IRSAD score (greater than median)	0.96 (0.81–0.99)	0.97 (0.93–1.01)
Prior percutaneous coronary interventions/coronary artery bypass graft	0.73 (0.67–0.80)	0.71 (0.65–0.78)
Atrial fibrillation/flutter	1.20 (1.15–1.26)	1.10 (1.05–1.15)
Prior valve replacement	0.99 (0.83–1.17)	—
Hypertension	0.97 (0.93–1.02)	—
Hyperlipidaemia	0.77 (0.69–0.85)	0.64 (0.57–0.70)
Charlson comorbidity index >1.0	1.16 (1.15–1.17)	1.21 (1.20–1.22)
**Year groups**		
2002–2005	1	1
2006–2009	0.93 (0.88–0.99)	0.87 (0.82–0.92)
2010–2013	0.71 (0.67–0.76)	0.65 (0.61–0.92)
2014–2017	0.57 (0.54–0.61)	0.48 (0.45–0.51)
2018–2021	0.49 (0.46–0.52)	0.41 (0.38–0.44)

CI = confidence interval; IRSAD = Index of Relative Social Advantage and Disadvantage.

* Univariable and multivariable predictors of in-hospital mortality was based on binary logistic regression analysis. Only variables with P<0.05 in the univariable analyses were included in the multivariable model.

Reference

1. Australian Bureau of Statistics. Statistical Area Level 2. Australian Statistical Geography Standard (ASGS) edition 3, July 2021 – June 2026. 20 July 2021. https://www.abs.gov.au/statistics/standards/australian-statistical-geography-standard-asgs-edition-3/jul2021-jun2026/main-structure-and-greater-capital-city-statistical-areas/statistical-area-level-2 (viewed Dec 2023).

STROBE statement

Page numbers refer to submitted manuscript, not the published article or its supporting material.

	Item No	Recommendation
**Title and abstract**	1	(*a*) Indicate the study's design with a commonly used term in the title or the abstract<br>**Page 1: Title page**
		(*b*) Provide in the abstract an informative and balanced summary of what was done and what was found<br>**Page 2: Abstract**
**Introduction**		
Background/rationale	2	Explain the scientific background and rationale for the investigation being reported<br>**Page 5: Introduction**
Objectives	3	State specific objectives, including any prespecified hypotheses<br>**Page 6: Introduction**
**Methods**		
Study design	4	Present key elements of study design early in the paper<br>**Pages 6 – 9: Methods.**
Setting	5	Describe the setting, locations, and relevant dates, including periods of recruitment, exposure, follow-up, and data collection<br>**Page 6: Study population, data sources**
Participants	6	(*a*) *Cohort study*—Give the eligibility criteria, and the sources and methods of selection of participants. Describe methods of follow-up<br>**Page 6: Study population**.
Variables	7	Clearly define all outcomes, exposures, predictors, potential confounders, and effect modifiers. Give diagnostic criteria, if applicable<br>**Page 7: Data sources and study outcome**.
Data sources/ measurement	8*	For each variable of interest, give sources of data and details of methods of assessment (measurement). Describe comparability of assessment methods if there is more than one group<br>**Page 8: Statistical analysis, Supplementary Table 3**
Bias	9	Describe any efforts to address potential sources of bias<br>**Page 16: Discussion (limitations)**
Study size	10	Explain how the study size was arrived at<br>**Not applicable**
Quantitative variables	11	Explain how quantitative variables were handled in the analyses. If applicable, describe which groupings were chosen and why<br>**Page 7 – 8: Data sources, Statistical analysis**
Statistical methods	12	(*a*) Describe all statistical methods, including those used to control for confounding<br>**Page 8: Statistical analysis.**
		(*b*) Describe any methods used to examine subgroups and interactions
		(*c*) Explain how missing data were addressed
		(*d*) *Cohort study*—If applicable, explain how loss to follow-up was addressed
		(*e*) Describe any sensitivity analyses
**Results**		
Participants	13*	(a) Report numbers of individuals at each stage of study—eg numbers potentially eligible, examined for eligibility, confirmed eligible, included in the study, completing follow-up, and analysed<br>**Results: Page 9, Supplementary Figure 1**
		(b) Give reasons for non-participation at each stage
		(c) Consider use of a flow diagram
Descriptive data	14*	(a) Give characteristics of study participants (eg demographic, clinical, social) and information on exposures and potential confounders<br>**Results: Page 9**
		(b) Indicate number of participants with missing data for each variable of interest
		(c) *Cohort study*—Summarise follow-up time (eg, average and total amount)

Outcome data	15*	*Cohort study*—Report numbers of outcome events or summary measures over time<br>**Results: Page 10**
Main results	16	*(a)* Give unadjusted estimates and, if applicable, confounder-adjusted estimates and their precision (eg, 95% confidence interval). Make clear which confounders were adjusted for and why they were included<br>**Results: Page 10 - 11**
		(*b*) Report category boundaries when continuous variables were categorized
		(*c*) If relevant, consider translating estimates of relative risk into absolute risk for a meaningful time period
Other analyses	17	Report other analyses done—eg analyses of subgroups and interactions, and sensitivity analyses<br>**Not applicable**
**Discussion**		
Key results	18	Summarise key results with reference to study objectives<br>**Discussion: Page 12**
Limitations	19	Discuss limitations of the study, taking into account sources of potential bias or imprecision. Discuss both direction and magnitude of any potential bias<br>**Discussion: Page 16**
Interpretation	20	Give a cautious overall interpretation of results considering objectives, limitations, multiplicity of analyses, results from similar studies, and other relevant evidence<br>**Discussion: Page 13 - 14**
Generalisability	21	Discuss the generalisability (external validity) of the study results<br>**Discussion: Page 13**
**Other information**		
Funding	22	Give the source of funding and the role of the funders for the present study and, if applicable, for the original study on which the present article is based<br>**Not applicable**

*Give information separately for cases and controls in case-control studies and, if applicable, for exposed and unexposed groups in cohort and cross-sectional studies.

Note: An Explanation and Elaboration article discusses each checklist item and gives methodological background and published examples of transparent reporting. The STROBE checklist is best used in conjunction with this article (freely available on the Web sites of PLoS Medicine at http://www.plosmedicine.org/, Annals of Internal Medicine at http://www.annals.org/, and Epidemiology at http://www.epidem.com/). Information on the STROBE Initiative is available at www.strobe-statement.org.
